# Tolerance of *Tetraselmis tetrathele* to High Ammonium Nitrogen and Its Effect on Growth Rate, Carotenoid, and Fatty Acids Productivity

**DOI:** 10.3389/fbioe.2021.568776

**Published:** 2021-01-28

**Authors:** Abd Wahab Farahin, Ikhsan Natrah, Norio Nagao, Fatimah Md. Yusoff, Mohamed Shariff, Sanjoy Banerjee, Tomoyo Katayama, Masatoshi Nakakuni, Mitsuhiko Koyama, Kiyohiko Nakasaki, Tatsuki Toda

**Affiliations:** ^1^Laboratory of Marine Biotechnology, Institute of Bioscience, Universiti Putra Malaysia, Serdang, Malaysia; ^2^Department of Aquaculture, Faculty of Agriculture, Universiti Putra Malaysia, Serdang, Malaysia; ^3^International Institute of Aquaculture and Aquatic Sciences, Universiti Putra Malaysia, Serdang, Malaysia; ^4^Bluescientific Shinkamigoto Co. Ltd., Nagasaki, Japan; ^5^Department of Veterinary Clinical Studies, Faculty of Veterinary Medicines, Universiti Putra Malaysia, Serdang, Malaysia; ^6^Graduate School of Agricultural and Life Sciences, The University of Tokyo, Bunkyo, Japan; ^7^Faculty of Agriculture, Kagawa University, Miki, Japan; ^8^School of Environment and Society, Tokyo Institute of Technology, Ookayama, Japan; ^9^Department of Environmental Engineering for Symbiosis, Faculty of Engineering, Soka Meguro University, Hachioji, Japan

**Keywords:** ammonium nitrogen tolerance, microalgae, photosynthetic efficiency, chlorophyll *a*, *Tetraselmis tetrathele*

## Abstract

Microalgae can use either ammonium or nitrate for its growth and vitality. However, at a certain level of concentration, ammonium nitrogen exhibits toxicity which consequently can inhibit microalgae productivity. Therefore, this study is aimed to investigate the tolerance of *Tetraselmis tetrathele* to high ammonium nitrogen concentrations and its effects on growth rate, photosynthetic efficiency (*F*_*v*_*/F*_*m*_), pigment contents (chlorophyll *a*, lutein, neoxanthin, and β-carotene), and fatty acids production. Experiments were performed at different ammonium nitrogen concentrations (0.31–0.87 gL^−1^) for 6 days under a light source with an intensity of 300 μmol photons m^−2^ s^−1^ and nitrate-nitrogen source as the experimental control. The findings indicated no apparent enhancement of photosynthetic efficiency (*F*_v_/*F*_m_) at high levels of ammonium nitrogen (${\text{NH}}_{4}^{+}$-N) for *T. tetrathele* within 24 h. However, after 24 h, the photosynthetic efficiency of *T. tetrathele* increased significantly (*p* < 0.05) in high concentration of ${\text{NH}}_{4}^{+}$-N. Chlorophyll *a* content in *T. tetrathele* grown in all of the different ${\text{NH}}_{4}^{+}$-N levels increased significantly compared to nitrate-nitrogen (NO_3_-N) treatment (*p* < 0.05); which supported that this microalgal could grow even in high level of ${\text{NH}}_{4}^{+}$-N concentrations. The findings also indicated that *T. tetrathele* is highly resistant to high ammonium nitrogen which suggests *T. tetrathele* to be used in the aquaculture industry for bioremediation purpose to remove ammonium nitrogen, thus reducing the production cost while improving the water quality.

## Introduction

In recent years, the rising growth and life expectancy of the global population had resulted in increased demand in energy, healthy food, water, drugs, and other resources. This has caught the attention of researchers worldwide to study the use of microalgae as a goal toward sustainable development. According to Rahman (2020), there is an increasing demand of microalgae-based products, as the global market for microalgae is projected USD 53.43 billion in the year 2026 as compared to USD 32.60 billion in 2017. These figures show that the microalgae industry is steadily growing and gaining more attention for extensive use in various sectors in the future. Research to identify inexpensive sources of nutrients element to mass culture microalgae is needed to reduce its cost of production to meet the increasing global market demand.

The enhancement of cultivation conditions through various techniques helps to contribute to the growth and production of numerous compounds in microalgae. Nitrogen, in the form of either ammonium (${\text{NH}}_{4}^{+}$) or nitrate (${\text{NO}}_{3}^{-}$), is an essential nutrient required for microalgae growth which subsequently contributes to the biomass produced. Ammonium is the most predominant source of nitrogen that exists in urban, agricultural and aerobic digested effluents with a wide variation in its concentrations, ranging from low (10 mgL^−1^-N) to high concentrations (2,000 mgL^−1^-N) (Cai et al., 2013; Krishna Reddy et al., 2017). It is also documented that certain level of ammonium nitrogen concentration is toxic and can inhibit microalgae productivity. Thus, further elucidation of microalgae ammonium nitrogen tolerance is needed, as some previous studies had pointed out that different strains of microalgae require different levels of nitrogen uptake (Raven et al., 1992; Feng et al., 2020).

Besides, Collos and Harrison (2014) reported that ammonium has both long-term (over days) and short-term (over minutes to hours) physiological effects on microalgae, such as growth rate, the efficiency of photosynthesis and other responses. Generally, these short-term responses could translate into long-term effects during the transient lag or induction phase, which subsequently impacts the expected outcomes of competition among species over a growth period of several days. Since the high concentration of ammonium can affect the physiological processes within the cell, therefore, continuous time-based monitoring should be conducted either in short-term or long-term period culture to evaluate the photosynthetic efficiency of the cells. Thus, the inhibition or tolerance effect of microalgae to the ammonium present in its growth medium should be investigated thoroughly for an initial, short-term physiological response, especially in terms of photosynthesis efficiency, after the microalgae are abruptly exposed to ammonium effluent sources either for a few hours (during lag phase) until several days of growth.

*Tetraselmis tetrathele*, which belong to the Chlorophyta phylum, is a green marine microalga widely used in aquaculture as feeds for marine lives, such as molluscs (Blanchard et al., 2008; Lu et al., 2017), crustacean larvae (Magnotti et al., 2016) and as a probiotic in fish (Grotkjær et al., 2016; Dittmann et al., 2020). The wide application of *T. tetrathele* could be attributed to the presence of numerous bioactive, biochemical compounds, such as polyunsaturated fatty acids (PUFA), polysaccharides, lipids, protein, enzymes and carotenoids in the microalgae cells (Tsai et al., 2016; Di Lena et al., 2019; Farahin et al., 2019; Schüler et al., 2020). From the culturing perspective, Chlorophyceae was noted to exhibit significant tolerance to high ammonium nitrogen concentration compared to other algae phylum, such as Cyanophyceae, Diatomophyceae, Dinophyceae, Prymnesiophyceae, and Raphidophyceae (Collos and Harrison, 2014).

Generally, there are four major research fields in microalgae biotechnology which consist of wastewater treatment, carbon dioxide sequestration, biofuel production and high value-added molecules production (Levasseur et al., 2020). However, to date, limited studies in the above mentioned research areas resulted in a distinct knowledge gap in understanding the effects of high ammonium nitrogen on *Tetraselmis* sp. algal growth, physiological responses and metabolites production. Therefore, this study was conducted with two aims: (1) to examine the growth rate and the photosynthesis efficiency (*F*_v_/*F*_m_) of *T. tetrathele* species at high concentrations of ${\text{NH}}_{4}^{+}$ medium and (2) to quantify the production of pigments and PUFA profile with ${\text{NH}}_{4}^{+}$ and ${\text{NO}}_{3}^{-}$ as nitrogen sources.

## Materials and Methods

### Microalgal Culture and Media Preparation

*Tetraselmis tetrathele* (West) Butcher (UPMC-A0011) was isolated from Port Dickson, Malaysia and cultured in natural seawater enriched with modified f/2 media (Guillard and Ryther, 1962; Guillard, 1975) which consisted of (per liter): 75 mg NaNO_3_; 5 mg of NaH_2_PO_4_·H_2_O; 3.15 mg FeCl_3_·6H_2_O; 4.36 mg Na_2_EDTA·2H_2_O; 0.18 mg MnCl_2_·4H_2_O; 0.22 μg ZnSO_4_·7H_2_O; 0.01 mg CoCl_2_·6H_2_O; 9.8 μg CuSO_4_·5H_2_O; 6.3 μg Na_2_MoO_4_·2H_2_O; 0.1 mg thiamine.HCl; 0.5 μg biotin; 0.5 μg cyanocobalamin. The pre-culture was grown in 1 L column reactor under 300 μmol photons m^−2^ s^−1^ of light intensity, aerated with 0.2 L min^−1^ air and 1–2% CO_2_ at 25°C. Three batch series of pre-culture were done by sub-culturing every week and samples were taken daily to monitor the *F*_*v*_*/F*_*m*_values of inoculated culture with optimum condition. Values between 0.6 and 0.8 indicate high potential photosynthetic performances which represent chlorophyll production in actively growing cells (Geel et al., 1997). After pre-culture in the exponential phase, the culture was sub-cultured with the initial cell density to ~0.1 gL^−1^ dry weight.

### Experimental Design on Growth Conditions With High Level of Nitrogen Sources

A schematic diagram of the cultivation systems used in this experiment is shown in Supplementary Figure 1. The nitrogen source of modified f/2 media was replaced by ammonium chloride (NH_4_Cl) with different concentrations of 0.31, 0.61, and 0.87 gL^−1^-N which initial concentrations of free ammonia were 0.77, 1.50, and 2.08 mM, respectively and 0.31 gL^−1^ of ${\text{NO}}_{3}^{-}$-N was used as the control (see Supplementary Table 1). Experiments were conducted with triplicate each. Treatments with ammonium were conducted with Tricine (N-[Tris(hydroxymethyl)-methyl]-glycine) buffer solution to maintain the concentration of free ammonia (NH_3_). During the initial growth, pH was maintained to 7.8 ± 0.1 throughout the experiment. Tricine and no Tricine controls were also tested with *T. tetrathele* and no growth inhibition due to Tricine was observed. Microalgae cultures were grown at 12:12 light/dark cycle (300 μmol photons m^−2^ s^−1^) in room temperature (25 ± 1°C). The aeration and the ventilation ports were equipped with 0.2 μm filters (Millipore) to prevent contamination or release of algae. The reactors were sparged with 0.2 L min^−1^ air with 1–2% CO_2_.

### Growth Parameter Analysis

The pH of the samples was closely monitored daily to ensure system stability and constant free ammonia concentration by using pH meter (B-712, HORIBA, Japan). Total ammonia concentration for each culture condition was measured using colorimetric method (APHA, 2012). To estimate the content of free ammonia concentration, ratio of free ammonia to total ammonia (NH_3_ %) was calculated according to Equation (1):

(1) $$\begin{array}{l} NH_{3}\;\left(\%\right)=\frac{100}{1+\left[H^{+}\right]}\times K_{a} \end{array}$$

Where *K*_*a*_ is the dissociation constant of ammonia, 4.36 × 10^−10^ at 25°C and 35 PSU (Khoo et al., 1977).

A cell suspension sample was filtered through a combusted glass fiber filter (GF/A, Whatman, UK) to determine the dry-cell weight. The cell pellet was washed three times with 0.5 M ammonium formate to remove soluble salts, dried at 60°C for 24 h and subsequently cooled to room temperature in a desiccator before weighing. Microalgae cells density were counted using light microscopy and haemacytometer-based counting. The optical density was measured using a spectrophotometer (DR 1900-01, Hach) at the wavelength 530 nm. Dry-cell weight, cell number, and optical density were measured daily and all the analyses were conducted in triplicate.

### Chlorophyll Fluorescence Analysis

A variable chlorophyll fluorescence was measured outside the bioreactor with a pulse amplitude modulated fluorometer (PAM, Walz, Water-Pam, Germany) to determine the effect of high ammonium concentration on the photosynthesis performance to the microalgal cells. The maximum photosynthetic efficiency (*F*_*v*_*/F*_*m*_) was obtained under actinic light. Subsamples for fluorescence analysis were taken and put in dark condition for 30 min at 25°C. After acclimatization, 3 mL of the samples were immediately transferred into 15 mm diameter quartz cuvette as described by Obata et al. (2009). Once stable maximum fluorescence yield in the dark-adapted state (*F*_*o*_) was reached, a saturating pulse of 1,200 μmol photons at 655 nm m^−2^ s^−1^ for 0.8 s was supplied to determine the maximum fluorescence yield (*F*_*m*_) after dark acclimation. The maximum photosynthetic efficiency of PS II (*F*_*v*_*/F*_*m*_) was calculated using Equation (2) as described by Schreiber et al. (1986):

(2) $$\begin{array}{l} \frac{F_{v}}{F_{m}}\;=\;\frac{\left(F_{m}-\;F_{o}\right)}{F_{m}} \end{array}$$

The monitoring process was done at 0, 1, 3, 6, 24 until 144 h.

### Cellular Photosynthethic Pigment Contents Quantification

Samples for analysis of the pigment were filtered through GF/A glass fiber filters (Whatman, UK) and stored at −20°C until further use. Cells collected on the filters (0.1–1.0 g-dw L^−1^) were extracted with 3 mL methanol, sonicated to break the cell walls and then kept in −4°C in darkness for 48 h. Cell extracts were filtered through a 0.22 μm filter (diameter 13 mm, Nylon, Thermo Scientific, USA) to eliminate glass fibers and cellular debris. All procedures for the extraction were conducted under subdued light to prevent photodegradation of the pigments. Tenth microliters of standards and samples were injected into Shimadzu Prominence-i high-performance liquid chromatography (LC 2030) using reversed phase column (2.1 × 150 mm inner diameter, 5 μm, C18, XBridge, Ireland). The HPLC flow rate was 0.5 mL/min; column temperature 40°C.

The HPLC conditions were performed using 80:20 (v/v) methanol and 0.5 M ammonium acetate as an eluent A and 70:30 (v/v) methanol and ethyl acetate as an eluent B. The gradient elution was performed as follows: initial conditions were 0% of eluent B until 24.9 min, followed by 100% of eluent B at 25 min, this proportion was maintained for 9 min. The column was then returned to the initial condition at 34.01 min and maintained the initial mobile phase until the end of the run at 39 min. Detection and identification were performed using a photodiode array detector (λ detection = 440, 450, 460, 465, 478, and 665 nm). The injection volume was 10 μL: two injections were performed for each sample and standard. The standard curve and the retention times were calibrated using chlorophyll *a*, neoxanthin, lutein and β-carotene standards in methanol at four different concentrations (500, 250, 100, 50 mg/mL). All samples were analyzed in triplicates and the results were expressed as milligram per gram dry weight biomass (mg/g-dw).

### Fatty Acid Methyl Ester (FAME)

Samples were filtered through GF/A, washed with 0.5 ammonium formate, lyophilised and kept in −20°C until subjected to fatty acid analysis. Preparation of fatty acid methyl ester from total lipid was performed according to the modified method of Bligh and Dyer (1959). Filtered samples were extracted in 3 mL chloroform: methanol (1:2, v/v) solution and sonicated for 15 min at 15°C. The extract was centrifuged at 4,000 × g for 8 min, and the supernatant was transferred into a new glass tube. The remaining residue was re-extracted three times and obtained extracts were pooled. Ultrapure water of 10 mL was added to remove impurities in the extract and separated by centrifugation for 10 min at 3,000 × g. One hundred microliters of and internal standard of heneicosane (C21) was added into the extract and dried up completely under nitrogen gas at 35°C. For transesterification, 1 mL of methylation mixture (methanol: acetyl chloride, 100:5, v/v) were added to the dry lipid fraction and heated at 100°C for 60 min. After cooling to room temperature, 2 mL of hexane was added and shaken for 5 s. The upper hexane phase was collected and dried up at 45°C under steam of nitrogen gas.

Then, the FAME was immersed in hexane quantified by an Agilent 6890 gas chromatograph-mass spectrometer (GCMS) (6890N GC/5973MS, Agilent Technologies, USA) equipped with a capillary column 30 m × 0.25 mm ID × 0.25 μm (Zebron ZB-Wax, USA). Helium was used as a carrier gas at a flow rate of 1.3 mL min^−1^ at 75.7 kPa head pressure. The injector temperature was programmed at 250°C and detector temperature at 255°C. The column temperature was initially set at 50°C for 1 min, then programmed to increase to 100°C at 10°C min^−1^, held for 5 min, and set at 240°C at 4°C min^−1^ for 15 min. Identification of the individual spectrum obtained was performed by comparison of mass spectra with the NIST libraries using data analysis software (Agilent MSD Productivity ChemStation). Identification of each fatty acid was conducted by comparison between the retention time and the mass spectrum of the standard, and quantified by comparing their peak area with that of the internal standard.

### Data Handling and Statistical Analysis

All data are shown as mean ± standard deviation (SD) of three determinations which *n* = 3. For dry weight, cell number, *F*_*v*_*/F*_*m*_ and pigments, repeated-measure one-way ANOVA followed by Tukey multiple comparison tests were used to compare between treatments (${\text{NO}}_{3}^{-}$-N and ${\text{NH}}_{4}^{+}$-N) during the study period. FAME percentages were arcsine transformed before the statistical analysis. A one-way ANOVA followed by a multiple comparison *post-hoc* test (Tukey) was used for FAME in order to compare between treatments. Statistical software SPSS (IBM SPSS Statistics, version 20) was used for the analysis. *P*-values < 0.05 were considered to be significant.

## Results and Discussion

### Growth Performance of *Tetraselmis tetrathele* Under Different Concentrations of Ammonium

**Figure 1:**
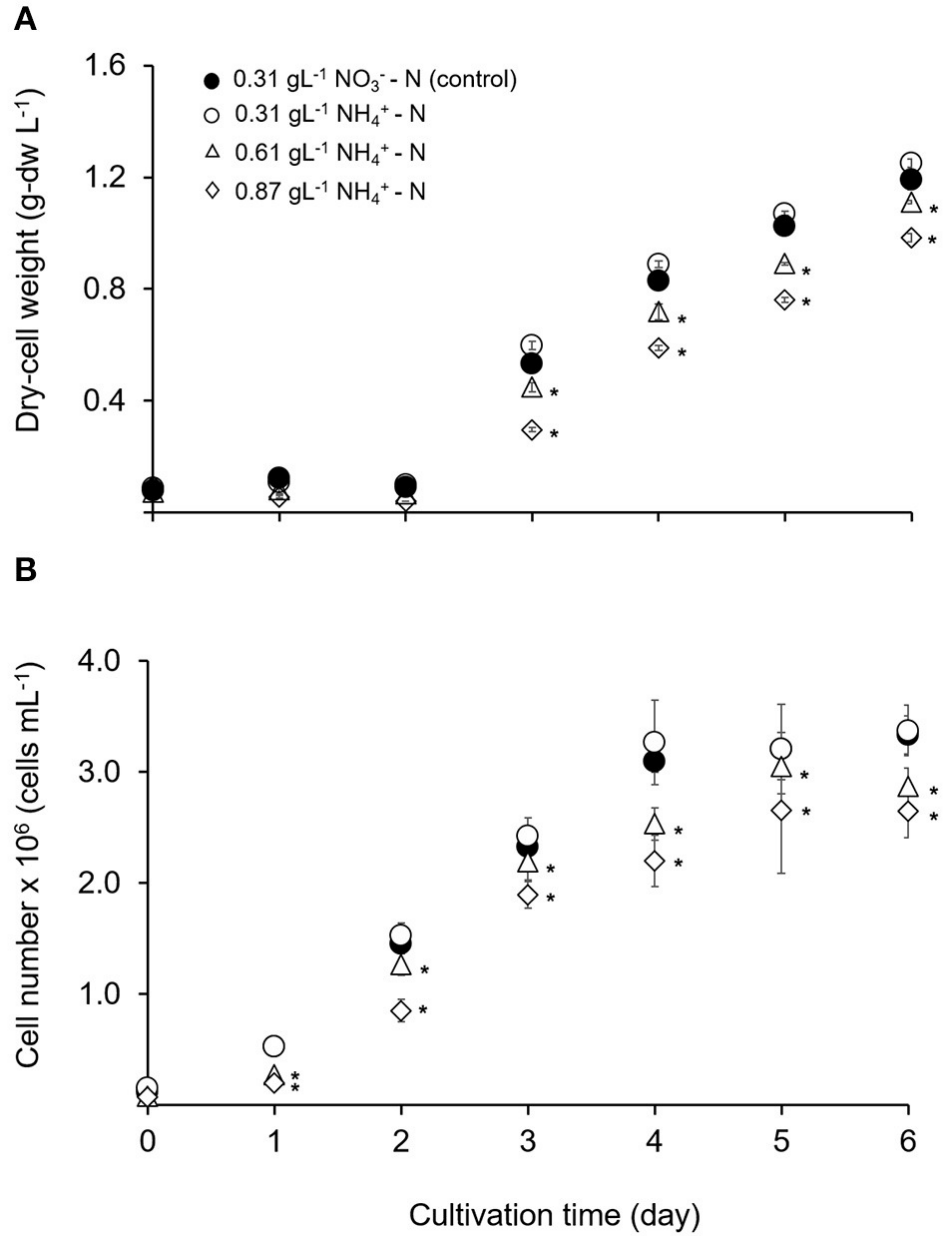
Time course of growth performance for *T. tetrathele* in different concentrations of ammonium nitrogen. **(A)** Dry-cell weight (g-dw L^−1^), and **(B)** cell number (cells mL^−1^). Points marked with an asterisk indicate significant differences between treatments on that specific day, *p* < 0.05. Values are means ± SD (*n* = 3).

The growth performance of *T. tetrathele* at different ammonium concentrations are shown in Figure 1. The algal dry-cell weight and cell number were increased with culturing time for ${\text{NH}}_{4}^{+}$-N conditions which were 0.31, 0.61, and 0.87 gL^−1^. Even though tested at the same nitrogen concentration (0.31 gL^−1^), the highest dry-cell weight and cell number with no significant differences to control (*p* > 0.05) were observed in ${\text{NH}}_{4}^{+}$-N condition with 1.25 ± 0.02 g-dw L^−1^ and 3.38 × 10^6^ cells mL^−1^, respectively. Meanwhile, the values of dry-cell weight and cell number under higher ${\text{NH}}_{4}^{+}$-N concentrations (0.61 and 0.87 gL^−1^) on day 6 were 1.1 and 1.2 times lower than those control conditions, 0.31 gL^−1^ ${\text{NO}}_{3}^{-}$-N (*p* < 0.05). From the representative results, although this microalgal tested in high concentrations, the cells were able to grow without inhibiting the growth.

Nitrogen is one of the primary essential nutrients required for algal growth and amino acids synthesis which is are the building blocks of proteins, beneficial in the formation of chlorophylls quintessential cellular machinery performing survival tasks, such as photosynthesis, light harvesting, and energy generation in microalgae (Grobbelaar, 2007). The inorganic nitrogen sources in microalgal cultivations are nitrate salts, nitrite salts and ammonium. Nitrate and nitrite salts are eventually converted to ammonium before being assimilated into amino acids via the glutamine synthetase/glutamate synthase pathway or the glutamate dehydrogenase pathway (Ramanna et al., 2014). This could be the reason that this microalgal showed higher dry-cell weight and cell number in 0.31 gL^−1^ ${\text{NH}}_{4}^{+}$-N condition compared to 0.31 gL^−1^ ${\text{NO}}_{3}^{-}$-N. Shi et al. (2000) reported similar result where *Chlorella protothecoides* absorbed ammonium and higher algal yields were obtained when nitrate was replaced by ammonium. Several other authors mentioned that ammonium was an excellent nitrogen source for certain strain of marine and freshwater algae (Dortch, 1990; Raven et al., 1992). Conversely, Feng et al. (2020) found that *Chlorella* sp. GN1 favored ${\text{NO}}_{3}^{-}$ where 11-fold significantly enhanced the growth rate compared to ${\text{NH}}_{4}^{+}$ as a nitrogen source. Thus, different algae species have different preference on type and level of concentration for the nitrogen sources (Zhuang et al., 2018).

However, ammonium nitrogen presents in two types: protonated cation ${\text{NH}}_{4}^{+}$ and gaseous form NH_3_ in aqueous solutions where the pH dictates the equilibrium between these two forms. Ammonium ion (${\text{NH}}_{4}^{+}$) dominates at pH < 9.25 meanwhile free NH_3_ (considered to be the most toxic form to the microorganisms) dominates above this pH, since the pK_a_ of ion equilibrium of ${\text{NH}}_{4}^{+}$/NH_3_ is 9.25 (Belkin and Boussiba, 1991; Drath et al., 2008). In the present study, the pH values were stable and maintained at 7.8 ± 0.1 due to the addition of Tricine buffer. This low pH values convert the free ammonia to the non-toxic ammonium ion and also considered 5% of free ammonia of the total ammonia concentration (Hargreaves and Tucker, 2004; Markou et al., 2014). Therefore, the possibility of change in free ammonia was very low since the pH, the temperature and the light intensity values were controlled.

Table 1 shows comparative studies on the evaluation of ammonium nitrogen activities cultivation in different strains of microalgae. Generally, Chlorophyceae showed high tolerant to high ammonium nitrogen concentration compared to other class, such as Cyanophyceae and Diatomophyceae and these findings were consistent with the conclusion of Collos and Harrison (2014). Besides that, Goto et al. (2018) studied on *Chlorella vulgaris* autotrophically grown in batch cultures of Walne's medium and tested the effect of ammonium on growth. It was found that the dry weight of *C. vulgaris* increased even under extreme high ammonium concentration of 0.96 g-N L^−1^ and reached around 4 g-dw L^−1^ of dry weight. In other situation, Katayama et al. (2020) found that after acclimatized in high ammonium concentration for a week, the two strains of *T. weissflogii* (TRG10-p103 and TRG10-p105) which belong to Diatomophyceae class, were able to grow up to 0.14 gL^−1^ ${\text{NH}}_{4}^{+}$-N. This could be the microalgae were responding to the stress and acclimatized, then successfully adapted to these conditions over the time (Borowitzka, 2018).

**Table 1:** Evaluation of ammonium nitrogen activities from microalgae.

**Species**	**Source of ${\text{NH}}_{4}^{+}$-N**	**${\text{NH}}_{4}^{+}$-N Con. (gL^**−1**^)**	**Light (μmol m^**−2**^s^**−1**^)**	**Temp. (^**°**^C)**	**pH**		**Buffer/** **pH adjust**	**CO_**2**_**	**Culture period**	**Max. dry-cell weight (gL^**−1**^)**	**Major observations**	**Study on lag phase**	**Photosynthesis efficiency (Yes/No)**	**References**
					**Initial**	**Final**								
*Chlorella* FACHB-1563 (Chlorophyceae)	Synthetic NH_4_Cl	0.26	70	25	9.25	10.5	–	°	5 h	–	- *F_*v*_/F_*m*_* dropped to 0.	Yes	Yes	Wang et al., 2019
*Chlorella* FACHB-1216 (Chlorophyceae)	Synthetic NH_4_Cl	0.26	70	25	9.25	10.5	–	°	5 h	–	- *F_*v*_/F_*m*_* dropped to 0.6 at 3 h cultivation and maintained until 5 h culture period.	Yes	Yes	Wang et al., 2019
*Nostoc* sp. strain H (Ge-Xian-Mi) (Cyanophyceae)	Synthetic NH_4_Cl	0.001	70	25	8.3 ± 0.2	8.3 ± 0.2	TAPS	°	4 days	–	- Chl *a* was 0.29 ± 0.07 mgL^−1^ - Value of *F_*v*_/F_*m*_* on day 4 was 0.31 ± 0.03 - Phycobiliprotein was 0.61 ± 0.07 mgL^−1^ - Saturating irradiance for photosynthesis and PS II activity decreased - Rapid fluorescence rise kinetics indicated oxygen-evolving complex of PS II was inhibitory site of ${\text{NH}}_{4}^{+}$	No	Yes	Dai et al., 2008
*Nostoc* sp. strain H (Ge-Xian-Mi) (Cyanophyceae)	Synthetic NH_4_Cl	0.003	70	25	8.3 ± 0.2	8.3 ± 0.2	TAPS	°	4 days	–	- Chl *a* was 0.07 ± 0.01 mgL^−1^ - Value of *F_*v*_/F_*m*_* on day 4 was 0.09 ± 0.03 - Phycobiliprotein was 0.16 ± 0.03 mgL^−1^ - Saturating irradiance for photosynthesis and PS II activity decreased - Rapid fluorescence rise kinetics indicated oxygen-evolving complex of PS II was inhibitory site of ${\text{NH}}_{4}^{+}$	No	Yes	Dai et al., 2008
*Nostoc* sp. strain H (Ge-Xian-Mi) (Cyanophyceae)	Synthetic NH_4_Cl	0.005	70	25	8.3 ± 0.2	8.3 ± 0.2	TAPS	°	4 days	–	- Chl *a* was 0.06 ± 0.01 mgL^−1^ - Value of *F_*v*_/F_*m*_* on day 4 was 0.04 ± 0.03 - Phycobiliprotein was 0.12 ± 0.07 mgL^−1^ - Saturating irradiance for photosynthesis and PS II activity decreased	No	Yes	Dai et al., 2008
											- Rapid fluorescence rise kinetics indicated oxygen-evolving complex of PS II was inhibitory site of ${\text{NH}}_{4}^{+}$			
*Nostoc* sp. strain H (Ge-Xian-Mi) (Cyanophyceae)	Synthetic NH_4_Cl	0.01	70	25	8.3 ± 0.2	8.3 ± 0.2	TAPS	°	4 days	–	- Chl *a* was 0.03 ± 0.01 mgL^−1^ - Value of *F_*v*_/F_*m*_* on day 4 was 0.06 ± 0.05 - Phycobiliprotein was 0.05 ± 0.02 mgL^−1^ - Saturating irradiance for photosynthesis and PS II activity decreased - Rapid fluorescence rise kinetics indicated oxygen-evolving complex of PS II was inhibitory site of ${\text{NH}}_{4}^{+}$	No	Yes	Dai et al., 2008
TRG10-p102 *Oocystis heteromucosa* (Chlorophyceae)	Synthetic NH_4_Cl	0.0196	150	25	8.0 ± 0.1	8.0 ± 0.1	HEPES	°	6 days	–	- The specific growth rate was not significantly different to control 0.0196 gL^−1^ ${\text{NO}}_{3}^{-}$-N	No	No	Katayama et al., 2020
TRG10-p102 *Oocystis heteromucosa* (Chlorophyceae)	Synthetic NH_4_Cl	0.042	150	25	8.0 ± 0.1	8.0 ± 0.1	HEPES	°	6 days	–	- The specific growth rate was not significantly different to control 0.0196 gL^−1^ ${\text{NO}}_{3}^{-}$-N	No	No	Katayama et al., 2020
TRG10-p102 *Oocystis heteromucosa* (Chlorophyceae)	Synthetic NH_4_Cl	0.084	150	25	8.0 ± 0.1	8.0 ± 0.1	HEPES	°	6 days	–	- The specific growth rate was not significantly different to control 0.0196 gL^−1^ ${\text{NO}}_{3}^{-}$-N	No	No	Katayama et al., 2020
TRG10-p102 *Oocystis heteromucosa* (Chlorophyceae)	Synthetic NH_4_Cl	0.140	150	25	8.0 ± 0.1	8.0 ± 0.1	HEPES	°	6 days	–	- The specific growth rate was not significantly different to control 0.0196 gL^−1^ ${\text{NO}}_{3}^{-}$-N	No	No	Katayama et al., 2020
TRG10-p103 *Thalassiosira weissflogii* (Diatomophyceae)	Synthetic NH_4_Cl	0.0196–0.140	150	25	8.0 ± 0.1	8.0 ± 0.1	HEPES	°	6 days	–	- The growth rate was inhibited cultured in >0.0196 gL^−1^ ${\text{NH}}_{4}^{+}$-N	No	No	Katayama et al., 2020
TRG10-p103 *Thalassiosira weissflogii* (Diatomophyceae)	Synthetic NH_4_Cl	0.0196–0.210	150	25	8.0 ± 0.1	8.0 ± 0.1	HEPES	°	6 days	–	- After acclimatized experiments were carried out, microalagal was able to grow even in 0.14 gL^−1^ ${\text{NH}}_{4}^{+}$-N	No	No	Katayama et al., 2020
TRG10-p105 *Thalassiosira weissflogii* (Diatomophyceae)	Synthetic NH_4_Cl	0.0196–0.140	150	25	8.0 ± 0.1	8.0 ± 0.1	HEPES	°	6 days	–	- The growth rate was inhibited, cultured in >0.0196 gL^−1^ ${\text{NH}}_{4}^{+}$-N	No	No	Katayama et al., 2020
TRG10-p105 *Thalassiosira weissflogii* (Diatomophyceae)	Synthetic NH_4_Cl	0.0196 −0.210	150	25	8.0 ± 0.1	8.0 ± 0.1	HEPES	°	6 days	–	- After acclimatized experiments were carried out, microalagal was e were able to grow even in 0.14 gL^−1^ ${\text{NH}}_{4}^{+}$-N	No	No	Katayama et al., 2020
TRG10-p201 *Amphora coffeiformis* (Diatomophyceae)	Synthetic NH_4_Cl	0.0196–0.140	150	25	8.0 ± 0.1	8.0 ± 0.1	HEPES	°	6 days	–	- The growth rate was inhibited, cultured in >0.0196 gL^−1^ ${\text{NH}}_{4}^{+}$-N	No	No	Katayama et al., 2020
*Chlorella vulgaris* (Chlorophyceae)	Synthetic NH_4_Cl	0.32	300	25	7.8 ± 0.1	7.8 ± 0.1	Tricine	°	25 days	4.2	- V/V_max_ almost 1.0 indicated microalgal high tolerance to ammonium nitrogen concentration. - Higher dry-cell weight and maximum area productivity was observed compared to ${\text{NO}}_{3}^{\;-}$-N	No	No	Goto et al., 2018
*Chlorella vulgaris* (Chlorophyceae)	Synthetic NH_4_Cl	0.64	300	25	7.8 ± 0.1	7.8 ± 0.1	Tricine	°	22 days	4.0	- V/V_max_ almost 1.0 indicated microalgal high tolerance to ammonium nitrogen concentration. - Higher dry-cell weight and maximum area productivity was observed compared ${\text{NO}}_{3}^{\;-}$-N	No	No	Goto et al., 2018
*Chlorella vulgaris* (Chlorophyceae)	Synthetic NH_4_Cl	0.96	300	25	7.8 ± 0.1	7.8 ± 0.1	Tricine	°	22 days	4.0	- V/V_max_ almost 1.0 indicated microalgal high tolerance to ammonium nitrogen concentration. - Higher dry-cell weight and maximum area productivity was observed compared ${\text{NO}}_{3}^{\;-}$-N	No	No	Goto et al., 2018
*Chlorella vulgaris* (Chlorophyceae)	Synthetic NH_4_Cl	1.60	300	25	7.8 ± 0.1	7.8 ± 0.1	Tricine	°	22 days	4.0	- V/V_max_ was decreased when free ammonia concentration >1.80 mM. - Higher dry-cell weight and maximum area productivity was observed compared ${\text{NO}}_{3}^{\;-}$-N	No	No	Goto et al., 2018
*Chlorella vulgaris* (Chlorophyceae)	Synthetic NH_4_Cl	1.60	300	25	8.4 ± 0.1	8.4 ± 0.1	Tricine	°	14 days	2.8	- V/V_max_ was decreased when free ammonia concentration >1.80mM. - Low dry-cell weight and maximum area productivity was observed compared to ${\text{NO}}_{3}^{-}$-N due to high pH.	No	No	Goto et al., 2018
*Spirulina platensis* (Cyanophyceae)	Synthetic NH_4_Cl	0.02	55	30	7.0	n.m	n.m	°	18 days	0.75	- Showed the most efficient nutrient removal within 6 days	No	No	Converti et al., 2006
*Spirulina platensis* (Cyanophyceae)	Synthetic NH_4_Cl	0.03	55	30	7.0	n.m	n.m	°	18 days	0.73	- Showed slow rate of nutrient removal (12 days)	No	No	Converti et al., 2006
*Spirulina platensis* (Cyanophyceae)	Synthetic NH_4_Cl	0.04	55	30	7.0	n.m	n.m	°	18 days	0.73	- Showed slow rate of nutrient removal (16 days)	No	No	Converti et al., 2006
*Monoraphidium* spp. SDEC-17 (Chlorophyceae)	Complex wastewater (CW)	0.173	90	25	8.52	9.0	n.m	°	16 days	1.29	- Removal efficiency of ammonium nitrogen in CW was 99.75% - CW not significantly influenced to lipid accumulation - Fatty acids profile quite similar that seen in biodiesel from palm oil resembles biodiesel from palm oil	No	No	Jiang et al., 2016
*Monoraphidium* spp. SDEC-17 (Chlorophyceae)	Complex wastewater (CW) + BG11	0.173	90	25	8.41	9.0	n.m	°	16 days	1.04	- Removal efficiency of ammonium nitrogen in CW + BG11 was 99.72% - Fatty acids profile quite similar seen in biodiesel from palm oil resembles biodiesel from palm oil - Showed efficient nutrient removal from CW + BG11	No	No	Jiang et al., 2016
*Scenedesmus obliquus* (Chlorophyceae)	Domestic wastewater	0.013	107.94	25	n.m	n.m	n.m	°	10 days	0.38	- Based on response surface methodology (RSM) under optimized conditions of light intensity, microalgal showed better nutrient removal and high growth with high chlorophyll and lipid compared to *Spirulina platensis*	No	No	Fan et al., 2020
*Spirulina platensis* (Cyanophyceae)	Domestic wastewater	0.013	53.97	25	n.m	n.m	n.m	°	10 days	0.33	- Based on response surface methodology (RSM) under optimized conditions of light intensity, microalgal showed rich in protein and carbohydrate compared to *Scenedesmus obliquus*	No	No	Fan et al., 2020
*Chlorella vulgaris* (Chlorophyceae)	Activated wastewater	0.04	60–70	n.m	7.1	n.m	HCl/NaOH	°	14 days	0.76	- Ratio of activated wastewater to microalgal (1:0.75) was optimum symbiotic algal-bacterial interactions. - This ratio showed the highest efficient nutrient removal from wastewater - The highest lipid yield and flocculation efficiency was observed.	No	No	Leong et al., 2018
*Chlorella sorokiniana* UKM2 (Chlorophyceae)	POME	0.05	269.86	25	7.0	6.8	–	°	7 days	1.10	- Potential microalgal-assimilable organic carbon source in POME - Achieved maximum CO_2_ uptake rate - Showed efficient nutrient removal from POME	No	No	Ding et al., 2020
*Coelastrella* sp. UKM4 (Chlorophyceae)	POME	0.05	269.86	25	7.0	6.8	–	°	7 days	0.92	- Potential microalgal-assimilable organic carbon source in POME - Showed efficient nutrient removal from POME	No	No	Ding et al., 2020
*Chlorella pyrenoidosa* UKM7 (Chlorophyceae)	POME	0.05	269.86	25	7.0	6.8	–	°	7 days	1.10	- Potential microalgal-assimilable organic carbon source in POME - Showed efficient nutrient removal from POME	No	No	Ding et al., 2020
*Tetraselmis tetrathele* (Chlorophyceae)	Synthetic NH_4_Cl	0.31	300	25	7.8 ± 0.1	7.8 ± 0.1	Tricine	°	6 days	1.25	- Through acclimation in high ${\text{NH}}_{4}^{+}$-N, *F_*v*_/F_*m*_* value of *T. tetrathele*'s cell increased significantly after 24 h (*p* < 0.05) - Chl *a* increased significantly compared 0.31 gL^−1^ ${\text{NO}}_{3}^{-}$ -N	Yes	Yes	This study
*Tetraselmis tetrathele* (Chlorophyceae)	Synthetic NH_4_Cl	0.61	300	25	7.8 ± 0.1	7.8 ± 0.1	Tricine	°	6 days	1.11	- Through acclimation in high ${\text{NH}}_{4}^{+}$-N, *F_*v*_/F_*m*_* value of *T. tetrathele*'s cell increased significantly after 24 h (*p* < 0.05) - Chl *a* increased significantly compared 0.31 gL^−1^ ${\text{NO}}_{3}^{-}$ -N	Yes	Yes	This study
*Tetraselmis tetrathele* (Chlorophyceae)	Synthetic NH_4_Cl	0.87	300	25	7.8 ± 0.1	7.8 ± 0.1	Tricine	°	6 days	0.98	- Through acclimation in high ${\text{NH}}_{4}^{+}$-N, *F_*v*_/F_*m*_* value of *T. tetrathele*'s cell increased significantly after 24 h (*p* < 0.05) - Chl *a* increased significantly compared 0.31 gL^−1^ ${\text{NO}}_{3}^{\;-}$-N	Yes	Yes	This study

*Temp., temperature; Max. dry-cell weight, maximum dry-cell weight; TAPS, N-[tris(hydroxymethyl)methyl-3-amino]propanesulfonic acid; HEPES, N-2-hydroxyethylpiperazine-N-2′-ethanesulfonic acid; Tricine, N-[Tris(hydroxymethyl)-methyl]-glycine; Con., concentration; POME, palm oil mill effluent; n.m., not mentioned*.

In respect to upscaling and future utilization in microalgae-based technologies, such as microalgae based wastewater treatment process, selection of suitable microalgal strain are required in order to tolerate in extreme conditions (aerobic digested effluents and agro-industrial wastewater) up to 2 gL^−1^-N (Cai et al., 2013; Krishna Reddy et al., 2017). Therefore, further elucidation on the physiological effect of this microalgal in ammonium nitrogen tolerance is needed and is discussed in the next section.

### Maximum Photosynthetic Efficiency (*F*_v_/*F*_m_)

**Figure 2:**
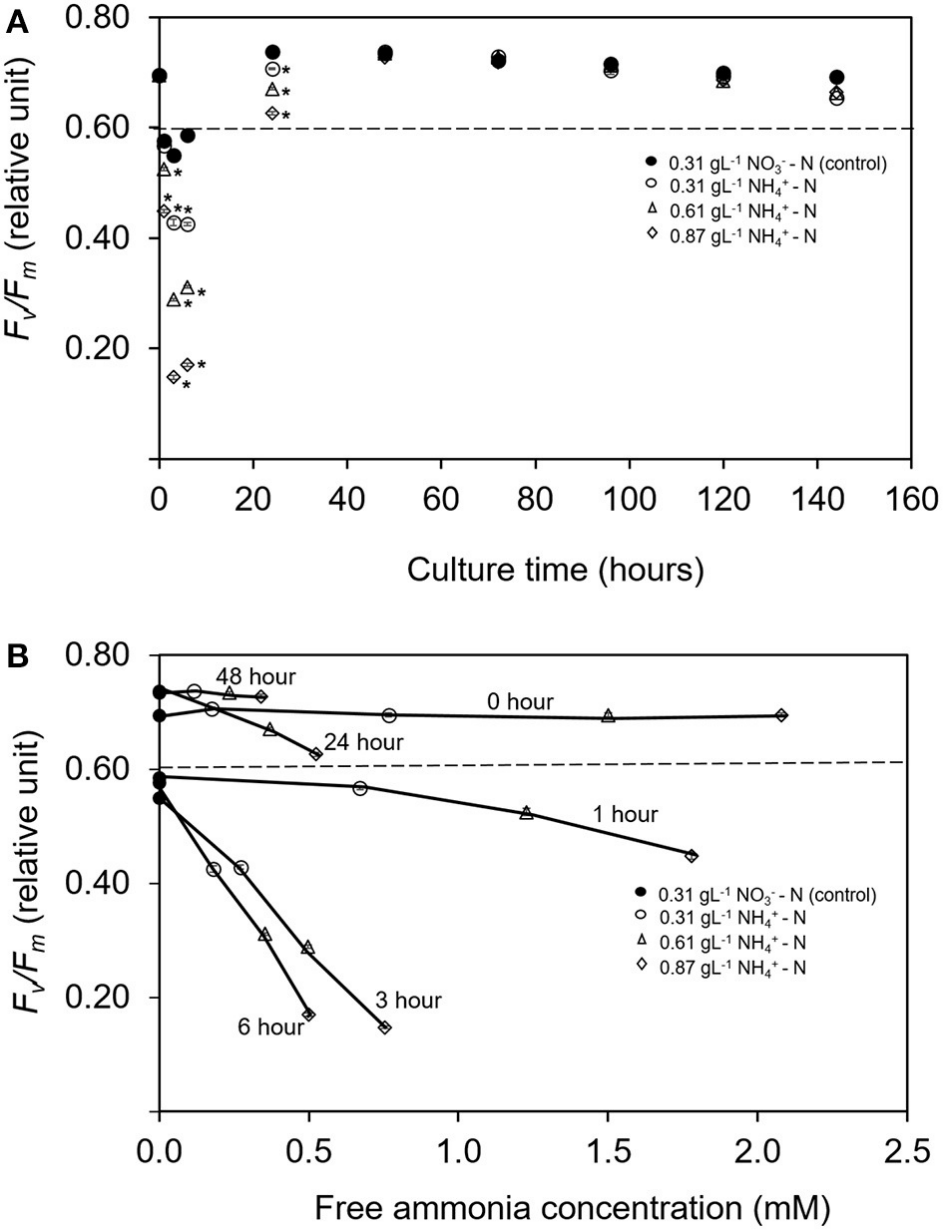
**(A)** Time course of maximum quantum yield of PS II (*F*_*v*_*/F*_*m*_) for *Tetraselmis tetrathele* during culturing period 144 h. **(B)** Relationship between relative *F*_*v*_*/F*_*m*_ with the ratio of free ammonia concentration for *T. terathele* within 48 h. Dotted lines indicate minimum value of healthy cell condition; *F*_*v*_*/F*_*m*_ (Geel et al., 1997; Masojídek et al., 2000). Points marked with an asterisk indicate significant differences between treatments on that specific day, *p* < 0.05. Values are means ± SD (*n* = 3).

Changes in *F*_v_/*F*_m_ is used as the diagnostic of photosynthetic health in microalgal cells (Geel et al., 1997; Cullen and Davis, 2003). Figure 2A illustrates the maximum photosynthetic efficiency (*F*_v_/*F*_m_) within 144 h in different ammonium nitrogen concentrations under experimental conditions. The value of *F*_v_/*F*_m_ sharply decreased in all treatments as early as hours. After 24 h, the value *F*_v_/*F*_m_ gradually increased to a stable condition with no significant differences for all treatments (*p* > 0.05). The relationship between *F*_v_/*F*_m_ and free ammonia to total ammonia concentration calculated from the pH and the total nitrogen under different time of culture period are shown in Figure 2B. Introduction of *Tetraselmis* in the column reactor at 0 h showed high value of *F*_v_/*F*_m_ (*p* < 0.05) even in high ammonium concentration (0.87 gL^−1^ ${\text{NH}}_{4}^{+}$-N). Nevertheless, at 1 h, the value of *F*_v_/*F*_m_ was the lowest (*p* < 0.005) at 0.4483, compared to the treatment of 0.61 and 0.31 gL^−1^ ${\text{NH}}_{4}^{+}$-N; with values at 0.5250 and 0.5670, respectively. After 3 h, the values of *F*_v_/*F*_m_ was again the lowest in the 0.87 gL^−1^ ${\text{NH}}_{4}^{+}$-N treatment with value of 0.1480 where the stress condition was maintained until 6 h. After 24 h of culture period, the cells were recovered where the value of *F*_v_/*F*_m_ increased higher than 0.6 and remained constant after 48 h with *F*_v_/*F*_m_ value at 0.730 ± 0.001. No significant difference was found among all treatments (*p* > 0.05). As mentioned by Gorai et al. (2014), the physiological health of microalgal cells can be diagnosed by changes in *F*_v_/*F*_m_. Values lower than 0.65 indicate that the cells are in physiological stress (Masojídek et al., 2000; Cullen and Davis, 2003) while higher values of *F*_v_/*F*_m_ indicate high potential photosynthetic performance for photosystem II (PS II) which represent chlorophyll production in actively growing cells.

Most of the previous studies focused on the interaction effect between microalgae growth and the addition of different ammonium dose exposure over certain time period, such as within a few minutes (Azov and Goldman, 1982), within a few hours (Collos and Slawyk, 2008) or effects in days after the addition of ammonium (Belkin and Boussiba, 1991; Tam and Wong, 1996). Nevertheless, no study has been reported on the intensive measurement on the growth rate reduction due to ammonium inhibition, especially in the first hours of cultivation. In the present study, the continuous decrease in *F*_v_/*F*_m_ was detected in the early stage of ammonium-enriched conditions. However, the cell was able to grow at a slow rate in lag-phase even though ammonium was used as nitrogen source in high concentration which was toxic. This slow/inhibition process was probably due to physiological change in nitrogen metabolism (Belkin and Boussiba, 1991; Vonshak and Torzillo, 2004; Drath et al., 2008; Markou et al., 2014). This process might suggest the ability to recover from PS II damage and was reflected in the increase of *F*_*v*_*/F*_*m*_ value leading to tolerance of *T. tetrathele'*s cell in high ammonium nitrogen concentration. Apart from that, modification in nitrogen metabolic pathway also affected the production of fatty acids as well as pigment production (Paliwal et al., 2017; Nayak et al., 2019) that will be discussed in the next section.

### Influence of Ammonium Nitrogen Availability on Pigments and Fatty Acid Profiles

**Figure 3:**
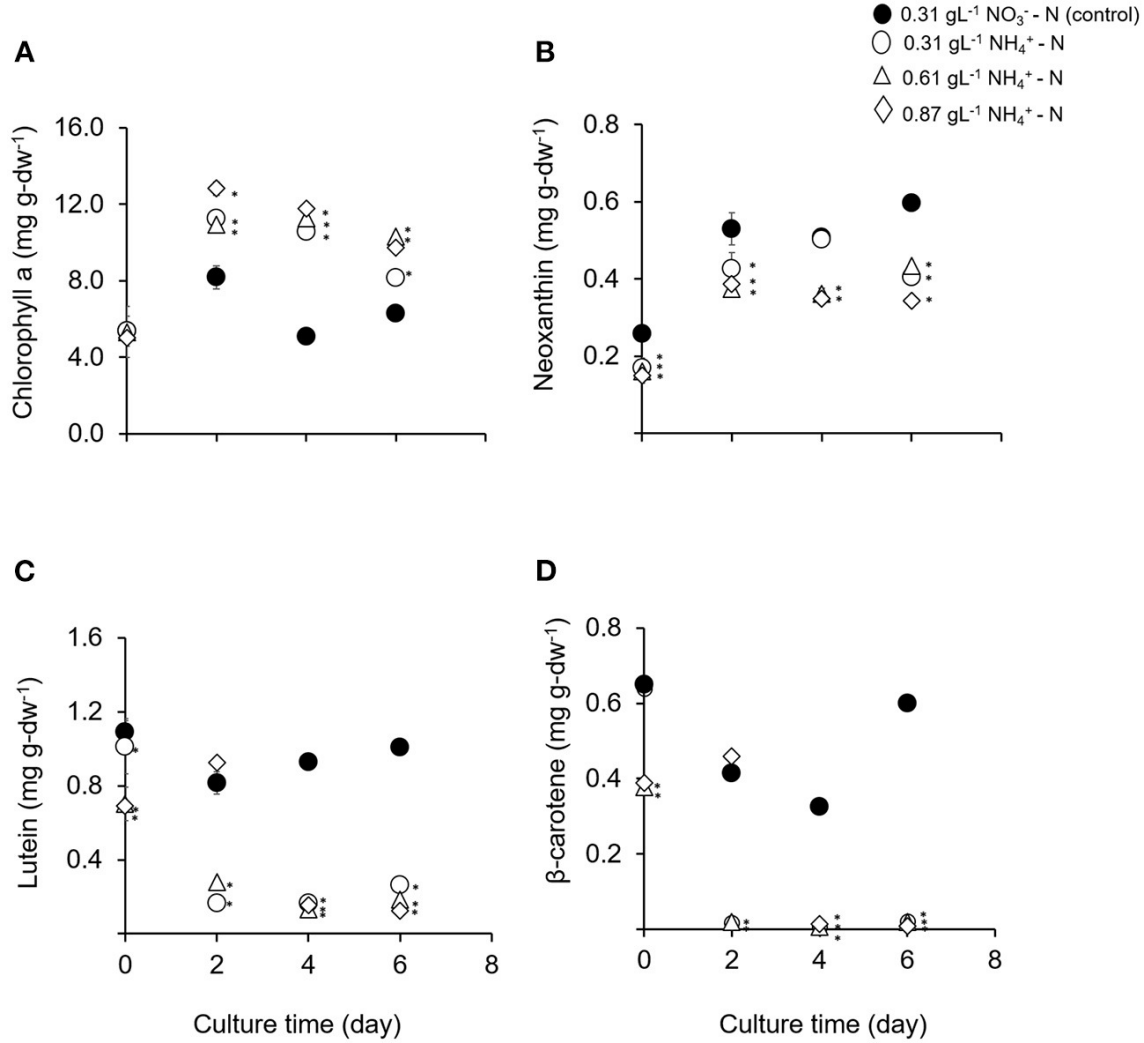
**(A–D)** Means and standard deviations of phototrophic pigment production in *T. tetrathele* by using different concentrations of ammonia as nitrate act as the control. Points marked with an asterisk indicate significant difference between treatments on that specific day, *p* < 0.05. Values are means ± SD (*n* = 3).

The effects of ammonium nitrogen in different concentration on pigments and fatty acids productivity were analyzed in this study. After day 2, the chlorophyll *a* content in all treatments was significantly higher compared to the control condition (*p* < 0.05) (Figure 3A). The dominant carotenoid contents were neoxanthin, lutein, and β-carotene. However, the concentration of these pigments was significantly lower compared to the control (*p* < 0.05) (Figures 3B–D). Chlorophyll *a* content is a good indicator for assessing the health of photosynthetic cells and is influenced by the type and the concentration of nitrogen sources (Piorreck et al., 1984; Baker and Oxborough, 2004). Meanwhile, carotenoids play fundamental roles as accessory pigments to protect themselves from photodamage and to aid photosynthesis (Nobel, 2009). Various environmental stresses make microalgae continuously tune their cellular mechanisms to cope with them. The accumulation of the stress metabolites is closely related to the changes occurring in their metabolic pathways (Ramos et al., 2008). Conversely in this study, the physiological activity and carotenoid compositions of *T. tetrathele* were not affected even in high ammonium concentrations.

Fatty acid methyl esters (FAME) profile of *T. tetrathele* grown in different concentration of ammonium nitrogen was analyzed to verify the quality of microalgae biomass (Table 2). The major fatty acids were C16:0 (palmitic acid), C18:1n-9 (oleic acid), C18:3n-6 (γ-linolenic acid, GLA), and C20:5n-3 (eicosapentaenoic acid, EPA), which comprised of 79–90% fatty acids. Similar results were obtained for *Tetraselmis* sp. by Abiusi et al. (2014), Das et al. (2016), Kim et al. (2016), and Tsai et al. (2016). This present study reported that treatment of 0.61 gL^−1^ ${\text{NH}}_{4}^{+}$-N contained the highest C_18_ fatty acids which was 65.5% followed by 0.31, 0.87 gL^−1^ ${\text{NH}}_{4}^{+}$-N, and control treatments. In addition, it was observed that *T. tetrathele* also produced 31.3% of PUFA even in 0.61 gL^−1^ ${\text{NH}}_{4}^{+}$-N. The highest ammonium concentration in this study resulted in the lowest concentration of PUFA (*p* < 0.05). On the other hands, 0.87 gL^−1^ ${\text{NH}}_{4}^{+}$-N conditions showed high MUFA contents compared to 0.31 and 0.61 gL^−1^ ${\text{NH}}_{4}^{+}$-N treatments (*p* < 0.05).

**Table 2:** Fatty acid composition (% of total fatty acids) of *Tetraselmis tetrathele* grown at day 6 in different concentration of nitrogen sources and review on two marine microalgae species.

**Lipid class**	**0.31 gL^**−1**^${\text{NO}}_{3}^{-}$-N (control)**	**0.31 gL^**−1**^${\text{NH}}_{4}^{+}$-N**	**0.61 gL^**−1**^${\text{NH}}_{4}^{+}$-N**	**0.87 gL^**−1**^${\text{NH}}_{4}^{+}$-N**	***Tetraselmis suecica***	***Tetraselmis* sp**.
C12:0	0.19 ± 0.10^a^	0.09 ± 0.00^c^	0.06 ± 0.00^d^	0.17 ± 0.00^b^	–	–
C14:0	0.46 ± 0.01^c^	0.63 ± 0.00^b^	0.38 ± 0.00^d^	1.17 ± 0.00^a^	0.49 ± 0.13	1.29 ± 1.03
C15:0	0.08 ± 0.00^c^	0.10 ± 0.00^b^	0.07 ± 0.00^d^	0.37 ± 0.00^a^	–	–
C16:0	36.60 ± 0.60^b^	31.92 ± 0.25^c^	19.29 ± 0.08^d^	47.85 ± 0.13^a^	16.39 ± 1.09	24.29 ± 0.33
C16:1n-7	4.32 ± 0.07^a^	0.42 ± 0.00^d^	2.35 ± 0.01^c^	2.46 ± 0.01^b^	–	1.70 ± 0.07
C16:2	1.04 ± 0.02^c^	1.32 ± 0.01^b^	0.87 ± 0.00^d^	1.79 ± 0.00^a^	0.87 ± 0.06	–
C16:3	0.58 ± 0.01^d^	4.23 ± 0.03^a^	1.30 ± 0.01^c^	2.51 ± 0.01^b^	3.93 ± 0.15	–
C17:0	0.12 ± 0.00^c^	0.25 ± 0.00^b^	0.10 ± 0.00^d^	0.59 ± 0.00^a^	–	4.36 ± 0.08
C17:1	n.d	n.d	0.04 ± 0.00	n.d	–	–
C17:3	n.d	0.20 ± 0.00	n.d	n.d	–	–
C18:0	0.83 ± 0.01^d^	1.34 ± 0.01^b^	1.09 ± 0.00^c^	2.76 ± 0.01^a^	–	0.43 ± 0.05
C18:1n-9	21.00 ± 0.34^d^	26.95 ± 0.21^c^	50.77 ± 0.21^a^	40.30 ± 0.11^b^	16.13 ± 0.56	18.00 ± 0.22
C18:2n-6	5.50 ± 0.09^a^	5.44 ± 0.04^b^	2.13 ± 0.01^c^	<0.001^d^	7.57 ± 0.11	11.39 ± 0.20
C18:3n-6	11.56 ± 0.19^b^	20.18 ± 0.16^a^	11.54 ± 0.05^b^	<0.001^c^	–	18.54 ± 0.21
C20:1n-9	2.09 ± 0.03^b^	2.14 ± 0.02^a^	1.10 ± 0.00^c^	n.d	–	–
C20:3n-3	3.00 ± 0.05	n.d	n.d	n.d	–	–
C20:4n-6	2.59 ± 0.04	n.d	n.d	n.d	1.48 ± 0.05	–
C20:5n-3	10.05 ± 0.16^a^	4.48 ± 0.03^c^	8.89 ± 0.04^b^	<0.001^d^	4.24 ± 0.06	–
C21:4	n.d	0.33 ± 0.00	n.d	n.d	–	–
C22:6n-3	<0.001^a^	<0.001^a^	<0.001^a^	<0.001^a^	–	–
Σ SFA	38.3 ± 0.25^b^	34.3 ± 0.20^c^	21.0 ± 0.04^d^	53.2 ± 0.04^a^	16.88 ± 0.22	37.00 ± 0.00
Σ UFA	61.7 ± 0.16^c^	65.7 ± 0.08^b^	79.0± 0.05^a^	47.0 ± 0.02^d^	86.52 ± 0.00	63.00 ± 0.00
Σ MUFA	44.4 ± 0.05^d^	44.9 ± 0.05^c^	68.7 ± 0.07^b^	90.8 ± 0.01^a^	25.89 ± 0.57	19.70 ± 0.00
Σ PUFA	55.6 ± 0.10^a^	55.1 ± 0.10^b^	31.3 ± 0.15^c^	9.2 ± 0.05^d^	57.23 ± 0.39	43.30 ± 0.00
Σ omega 6	19.65 ± 0.04^b^	25.62 ± 0.05^a^	13.67 ± 0.11^c^	0.03 ± 0.01^d^	9.05 ± 0.00	11.39 ± 0.20
Σ omega 3	13.04 ± 0.05^a^	4.48 ± 0.04^c^	8.89± 0.08^b^	0.01 ± 0.00^d^	4.24 ± 0.06	18.54 ± 0.29
ω6/ω3	1.51 ± 0.06^d^	5.72 ± 0.06^a^	1.54± 0.02^c^	1.90 ± 0.17^b^	2.13 ± 0.00	0.61 ± 0.00
References	This study	This study	This study	This study	[a]	[b]

*n.d., not detected. Data refer to the end of culture period*.

a−d *Results presented as mean ± SD (n = 3). Different letters within the same row indicates significant difference (p < 0.05). Σ SFA = total saturated fatty acid, Σ UFA = total unsaturated fatty acid, Σ MUFA = total mono-unsaturated fatty acid, Σ PUFA = total polyunsaturated fatty acid, ω is omega. [a] Abiusi et al. (2014), [b] Kim et al. (2016)*.

The variation of fatty acid profile indicated microalgae accumulated lipids or starch as a crucial part of their survival mechanisms which was also reported by Paliwal et al. (2017). In this regards, 0.87 gL^−1^ ${\text{NH}}_{4}^{+}$-N had 53.2% of saturated fatty acids which was the highest compared to other treatments (*p* < 0.05). When the microalga was exposed to extreme stress level conditions, as in 0.87 gL^−1^ ${\text{NH}}_{4}^{+}$-N, the unsaturated fatty acids tend to undergo oxidative cleavage resulting to higher degree of saturation in the microalgae lipids. Similar results were observed in nitrogen depletion conditions of *Chlorella vulgaris* and *Dunaliella tertiolecta* which led to increased degree of saturation (Stephenson et al., 2010; Lee et al., 2014). PUFA are the predominant fatty acids in the composition of structures in chloroplast membranes. PUFA play important roles to maintain the membrane functions including thermal adaptation, regulation of membrane fluidity and permeability, and also oxygen and electron transportations in cellular and tissue metabolisms (Chia et al., 2013; Lee et al., 2014). In this study, high concentration of ammonium nitrogen caused metabolic imbalance in the early stage of stress period and different fatty acid compositions were observed in each ammonium conditions. These results suggest that the ammonium stress could change the fatty acids profile and the media composition can control the type of fatty acids in the cell for production purposes. Stress response and adaptive process are associated with the photosynthetic apparatus. The high degree of saturation indicated the *T. tetrathele*'s cell undergone oxidative cleavage in order to protect themselves as a survival mechanism. As a result, the chlorophyll *a* in all ammonium treatments were high after recovering process and the carotenoid content was low compared to the control condition.

In the present study, the *T. tetrathele* strain rich in total UFA, MUFA, and PUFA could also benefit aquaculture organisms since PUFA provide essential fatty acids for the growth and the development of several species during early developmental stages including larvae, mollusc, and young aquatic organisms (Otero and Fábregas, 1997).

## Conclusion

This is an elemental work dealing with the adaptation of *T. tetrathele* to three different high level ammonium (${\text{NH}}_{4}^{+}$) concentrations by using nitrate (${\text{NO}}_{3}^{-}$) as the control. This microalgal showed the ability to recover from the damage of the photosynthetic apparatus of photosystem II and was able to grow without inhibiting the growth. The results of chlorophyll *a* content showed that all ${\text{NH}}_{4}^{+}$-N treatments were significantly higher compared to nitrate-nitrogen (NO_3_-N). Treatment in 0.87 gL^−1^ ${\text{NH}}_{4}^{+}$-N had 53.2% of saturated fatty acids which was the highest compared to other treatments and indicated the microalgal response to environmental conditions, such as media, as a crucial part of their survival mechanisms. This species is a valuable candidate to be used for mass culture for future microalgae-based technologies whereby can stimulate the economic activity toward attaining high income.

## Data Availability Statement

The raw data supporting the conclusions of this article will be made available by the authors, without undue reservation.

## Author Contributions

AF, IN, and NN conceived and designed the experiments, performed the experiments, analyzed the data, wrote the paper, prepared the figures and/or tables, and reviewed the drafts of the paper. MS contributed to the funding acquisition, reviewed, and edited the draft prior to the submission. SB, TK, MN, and MK were involved in the investigation of the study. FY, KN, and TT contributed to the funding acquisition of the research. All authors contributed to the article and approved the submitted version.

## Conflict of Interest

NN was employed by the company Bluescientific Shinkamigoto Co. Ltd. The remaining authors declare that the research was conducted in the absence of any commercial or financial relationships that could be construed as a potential conflict of interest.
