# Supplementary material for: Tolerance of Tetraselmis tetrathele to High Ammonium Nitrogen and Its Effect on Growth Rate, Carotenoid, and Fatty Acids Productivity
Source: Front Bioeng Biotechnol. 2021 Jan 28;9:568776. doi: 10.3389/fbioe.2021.568776 (PMC7876249; doi:10.3389/fbioe.2021.568776)
Supplement: Supplementary file 1 [file Data_Sheet_1.pdf]

Supplementary Material

TABLE S1 | Different concentration of nitrogen sources and pH in culture media at time 0

Nitrogen source	TAN (mM)	Nitrogen concentration (gL^{-1})	Free ammonia concentration (mM)	pH
$NaNO_3$ (control)	21.80	0.31	0.00	7.89
NH_4Cl	21.80	0.31	0.77	7.88
NH_4Cl	43.60	0.61	1.50	7.87
NH_4Cl	61.77	0.87	2.08	7.86

TAN = Total ammonia nitrogen (free ammonia, NH_3 + ammonium ion, NH_4^+)

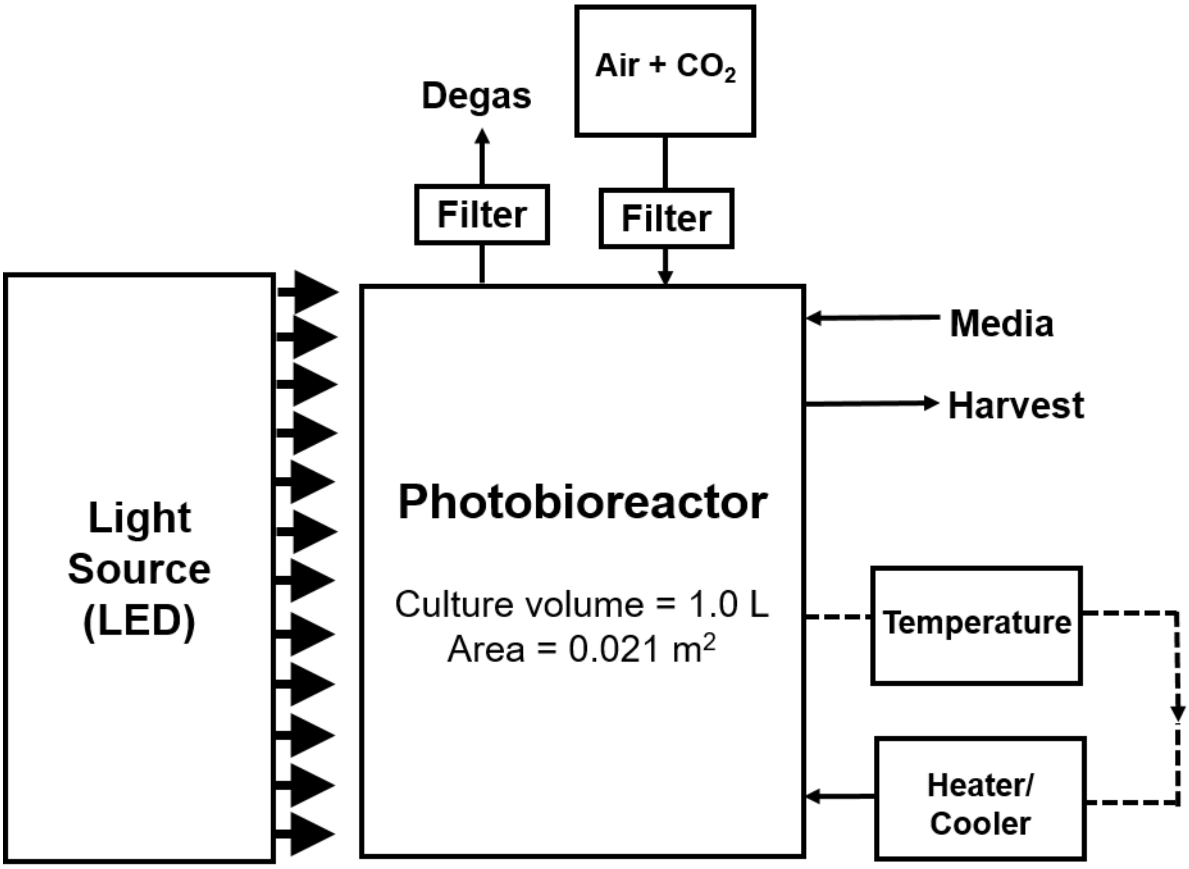

FIGURE S1 | Schematic diagram of cultivation system.
